# Triceps-sparing approach for open reduction and internal fixation of neglected displaced supracondylar and distal humeral fractures in children

**DOI:** 10.1007/s10195-015-0334-2

**Published:** 2015-01-22

**Authors:** Ahmed Shawkat Rizk

**Affiliations:** 1 Orthopaedics and Traumatology Department, Faculty of Medicine, Benha University, Benha, Egypt; 2 Shebeen el-kanater, Qualiobia Egypt

**Keywords:** Neglected distal humeral fractures, Children, Triceps-sparing approach, Kirschner wire fixation

## Abstract

**Background:**

Supracondylar humeral fractures are one of the most common skeletal injuries in children. In cases of displacement and instability, the standard procedure is early closed reduction and percutaneous Kirschner wire fixation. However, between 10 and 20 % of patients present late. According to the literature, patients with neglected fractures are those patients who presented for treatment after 14 days of injury. The delay is either due to lack of medical facilities or social and financial constraints. The neglected cases are often closed injuries with no vascular compromise. However, the elbow may still be tense and swollen with abrasions or crusts. In neglected cases, especially after early appearance of callus, there is no place for closed reduction and percutaneous pinning. Traditionally, distal humeral fractures have been managed with surgical approaches that disrupt the extensor mechanism with less satisfactory functional outcome due to triceps weakness and elbow stiffness. The aim of this study is to evaluate the outcome of delayed open reduction using the triceps-sparing approach and Kirschner wire fixation for treatment of neglected, displaced supracondylar and distal humeral fractures in children.

**Materials and methods:**

This prospective study included 15 children who had neglected displaced supracondylar and distal humeral fractures. All patients were completely evaluated clinically and radiologically before intervention, after surgery and during the follow-up. The follow-up period ranged from 8 to 49 months, with a mean period of 17 months. Functional outcome was evaluated according to the Mayo Elbow Performance Index (MEPI) and Mark functional criteria.

**Results:**

All fractures united in a mean duration of 7.2 weeks (range 5–10 weeks) with no secondary displacement or mal-union. Excellent results were found at the last follow-up in 13 of the 15 patients studied (86.66 %), while good results were found in two patients (13.33 %) according to the MEPI scale. According to the Mark functional criteria, there was one patient with a fair result (6.66 %).

**Conclusion:**

The results were very satisfactory if compared with traditional operative techniques, with many advantages including anatomical reduction and fixation of the fractures, avoidance of ulnar nerve injury, preservation of the extensor mechanism, decrease in incidence of myositis ossificans around the elbow and decrease in post-operative stiffness.

**Level of evidence:**

IV.

## Introduction

Supracondylar humeral fractures (SCHF) are common pediatric injuries [1] representing about 3 % of all fractures, and are the most common elbow fractures in children [2]. Over the past several decades there has been a shift from non-operative management to surgical stabilization for these fractures [3].

SCHF are classified using the modified Gartland classification and most of them are of extension type [4]. Displaced SCHF are challenging injuries to treat and entail technically difficult procedures for orthopedic surgeons [5, 6]. Supracondylar humeral fractures are usually treated as an emergency in children [3].

Currently, the preferred approach for the treatment of displaced pediatric supracondylar fractures is closed reduction and percutaneous pinning; this technique requires experience and is not free of complications or partial failure [7]. It fails in up to 25 % of patients [8], and requires remanipulation because of inadequate reduction or malpositioning of wires in 1–7 % of patients [9].

If attempts at closed reduction fail, then open reduction of the fracture followed by cross-pinning should be considered. Open reduction may also frequently be required in late-presented SCHF [3]. Severe swelling or skin problems around the elbow are the universally accepted indications for delaying surgical intervention following a SCHF in children. In developing countries, problems relating to disorganized health insurance systems and some traditional incorrect interventions (by non-medical personnel) unique to that specific country can also significantly influence the time interval between the injury and the definitive treatment. Under these circumstances, management of a late-presented SCHF becomes inevitable for the orthopedic surgeon [3].

Surgical exposure can be accomplished by a variety of approaches [10, 11]. A surgical approach should permit a safe and rapid reduction, with full anatomical alignment, obtaining adequate functional and cosmetic outcomes, as well as fewest complications. There is no clear evidence in the literature regarding which of the surgical approaches brings about the best functional and cosmetic outcomes, as well as minimizing complications [2].

## Materials and methods

This prospective study was carried out in the Orthopaedic Department at Benha University Hospital, Benha, Egypt from March 2007 to April 2013, and comprised 15 patients, all male. Their ages ranged from 6 to 11 years (mean 8.6 years). All patients had an initial treatment in the form of closed reduction and above elbow slab in private clinics or local hospitals and presented to the author late. The duration between their initial injury and presentation ranged from 16 to 34 days (mean 19 days). Twelve patients (80 %) had neglected extension type supracondyar fractures, two patients (13.33 %) had neglected flexion-type supracondylar fractures, while the last patient (6.67 %) had a neglected lower fourth humeral fracture. At the time of presentation, two patients (13.33 %) had skin abrasions and crusts with signs of radial nerve involvement in one patient and ulnar nerve involvement in the other. No patients had vascular involvement. No patients had open supracondylar fractures. No patients had any previous trial of surgical intervention in the form of closed reduction with percutaneous pinning.

Any case of acute displaced supracondylar fracture or supra-intercondylar fracture of the humerus or supracondylar fracture of the humerus after closure of the epiphysis was excluded. Only patients with neglected, displaced, supracondylar or lower humeral fractures with no previous operative intervention were included in this study.

**Fig. 1:**
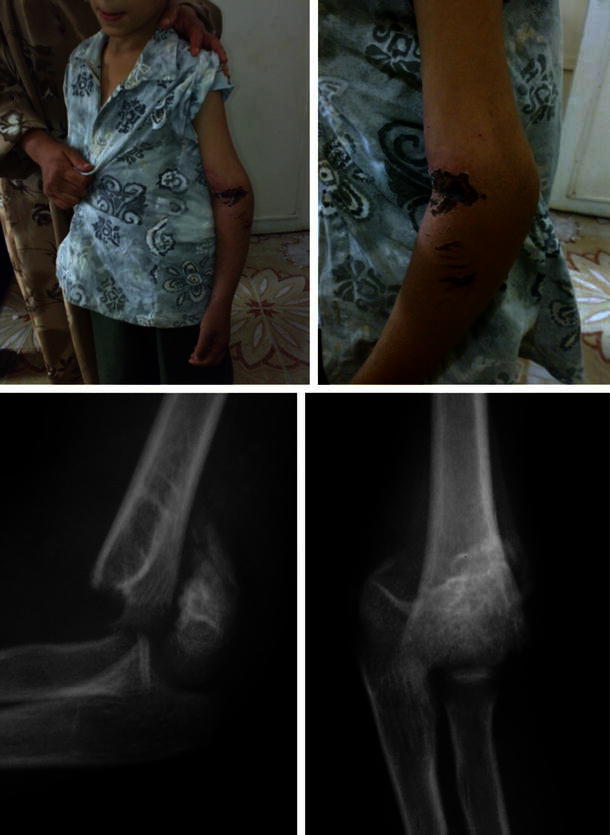
(*Top*) clinical photograph showing elbow swelling, deformity with sagging and crusts in a case of extension supracondylar fracture humerus neglected for 27 days. (*Bottom*) X-ray showing off-ending of the fragments with marked posterior displacement with early callus formation. Nerve conduction study showed incomplete radial nerve involvement (patient 1)

**Fig. 2:**
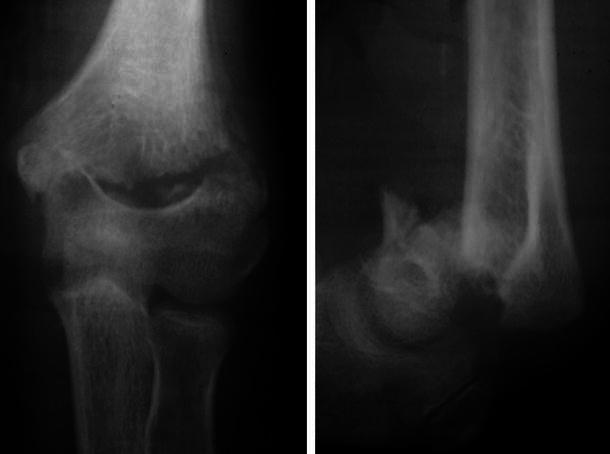
X-ray of a patient with flexion-type supracondylar fracture humerus neglected for 22 days showing off-ending of the fragments with marked anterior displacement of the distal stump with early callus formation (seen in antero-posterior view) (patient 14)

**Fig. 3:**
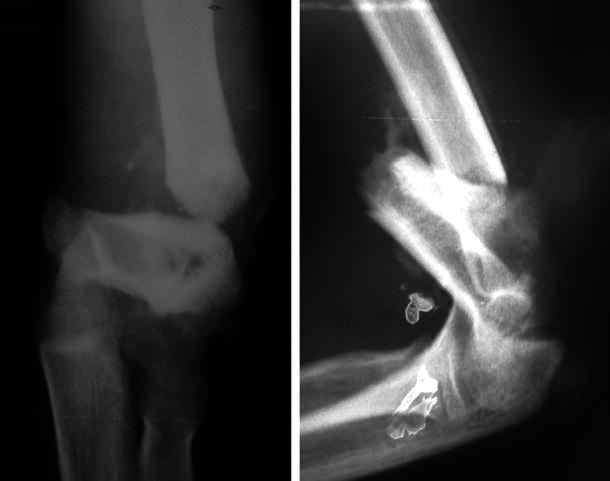
X-ray of a patient with displaced lower fourth fracture humerus neglected for 34 days showing off-ending of the fragments with visible callus formation in the lateral view (patient 8)

Careful evaluation of the patients was made pre-operatively; a complete history of the initial trauma was taken, with the associated injuries, the initial management done, and the reason why the parents sought advice. The interval between initial injury and presentation was recorded. Examination included careful inspection of the skin and soft tissue envelope around the elbow, deformity or sagging at the elbow region (Fig. 1) and active finger movements. Palpation was performed for distal pulse and tenderness over the elbow. Pre-operative radiographs were evaluated for the presence of comminution, other associated injuries around the elbow that may affect the treatment or the prognosis, early callus formation, the site of the fracture and its type as extension- or flexion-type injuries (Figs. 1, 2, 3).

Nerve conduction studies were done to document neural insult prior to intervention in any patient suspected to have nerve injury. All the patients in this study were treated by open reduction and internal fixation (ORIF) through a posterior triceps-sparing approach using at least two crossing wires depending on the size of the distal fragment and the intra-operative stability.

The procedure was done under general anesthesia, with tourniquet applied. All the patients were in the prone position with their arms supported on a side post with the elbow semi-flexed to relive tension on the ulnar and radial nerves. A midline straight skin incision was made with the proximal two-thirds of the incision above the tip of the olecranon while the remaining one-third was over the back of the forearm from the tip of the olecranon. The distal part facilitates exposure and isolation of the ulnar nerve which is critical for the safe exposure of the distal humerus.

After exposure and isolation of the ulnar nerve, the scalpel was used to sharply separate the anterio-medial border of the triceps muscle from the medial intermuscular septum down to the bone.

Sharp dissection down to the bone was also done laterally between the anterio-lateral border of the triceps muscle and the lateral intermuscular septum with the radial nerve and the profunda brachii artery passed within it from the back of the arm anteriorly [12], so that the back of the humerus could be safely reached without endangering these vital structures.

By elevation and retraction of the whole bulk of the muscle, the posterior surface of the humerus could be safely reached without interruption or violation of the integrity of the triceps muscle and its tendon.

Manipulation and reduction of the displaced bony fragments, whatever the level of the fracture (supracondylar or lower fourth humeral fractures) or the direction of displacement (anterior or posterior displacement of the distal fragment), could now be done easily and safely.

Anatomical reduction could easily be done and assessed both by palpation and by direct vision of the fracture with no need for image intensification. Fixation could then be achieved by at least two crossing Kirschner wires inserted under direct vision, avoiding the ulnar nerve and engaging the opposite cortex.

**Fig. 4:**
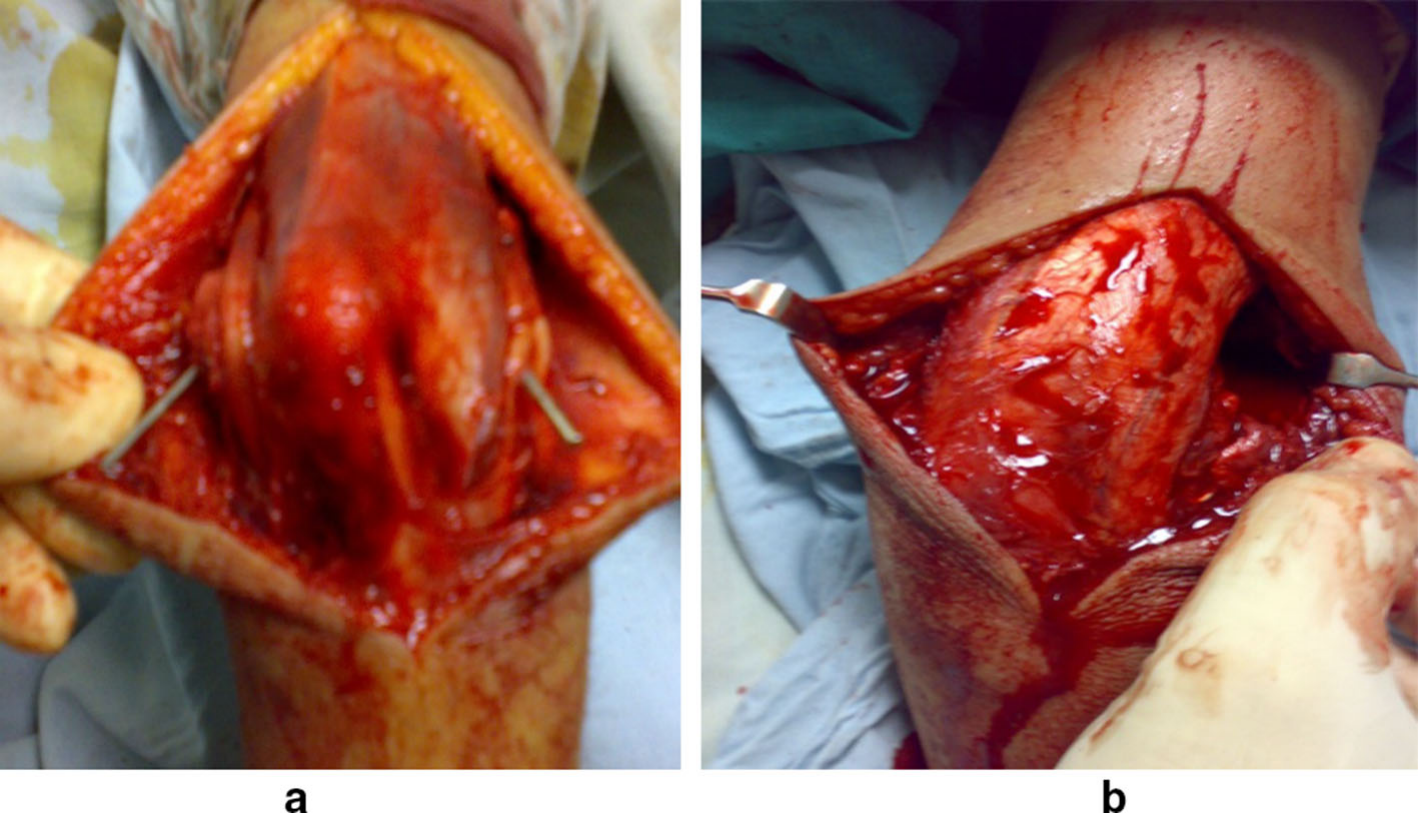
**a** The reduced fracture under the completely undisturbed triceps muscle was fixated by two crossing wires with the medial one distant from the identified ulnar nerve. After torniquet release, good hemostasis was achieved with the wires left outside the skin for easy removal. **b** The same procedure was done with the wires bent and kept inside the wound in the case of neglected displaced lower fourth humeral fracture

In all the patients with neglected supracondylar fractures, the wires were left protruding from the skin for easy removal as an outpatient procedure (Fig. 4a). In the case of lower fourth humeral fracture and due to the location of the fracture and age of the patient, the wires were impeded and kept within the wound to be left safely for a longer duration until union (Fig. 4b).

The tourniquet was removed before wound closure with good hemostasis, then the wound closed in layers (the subcutaneous and skin layers) with no need to insert a suction drain. An above elbow slab in 90° flexion was applied with no risk of edema or compartmental syndrome, as all patients were neglected for more than 2 weeks and there was no vascular insult in any patient; in addition a good hemostasis was achieved after torniquet removal before closure of the wound.

Post-operative care started immediately after recovery from anesthesia by evaluating the movements of all fingers and the neurovascular condition of the patient. Post-operative X-rays were taken to evaluate the result of intervention and to be used as a reference in the follow-up period to detect any position change or re-displacement. The above elbow slab and sutures were removed 2 weeks after the operation with active range of motion (ROM) started with the wires in place. Regular follow-up (clinical and radiological) was done every 2 weeks until complete union and wire removal, then monthly until complete restoration of the ROM and every 6 months subsequently until the last visit. The functional results were assessed according to Mark et al. [13] and the Mayo Elbow Performance Index (MEPI), which comprises four parameters: pain, arc of motion, stability, and activities of daily living.

Functional outcome was evaluated according to the MEPI as described by Turchin et al. [14]. MEPI is a four-part scale where clinical information is rated based on a 100-point scale, as follows:

- 90–100: excellent
- 75–89: good
- 60–74: fair
- Below 60: poor

1. Pain: The therapist asks the patient how severe the pain is and how frequently the pain appears. 45 points are for patients who do not have pain, 30 points are given to patients who have mild pain, and moderate pain results in 15 points; patients with severe pain get 0 points.
2. The arc of elbow motion: 20 points are given when the arm reaches more than 100° flexion; when the angle is between 100° and 50° the patient is given 15 points. When the maximum flexion is no more than 50°, then 5 points are given.
3. Stability: When the elbow is considered stable, 10 points are scored. A mildly unstable elbow results in 5 points. An unstable elbow receives 0 points.
4. ADL (Activities of Daily Living): five ADLs are each given 5 points, viz. combing hair, performing personal hygiene, eating, and putting on shirt and shoes.

The Mark et al. [13] scale also has four parts rated as excellent/good/fair/poor based on the following items: loss of motion, loss of the carrying angle and pain/neurovascular lesion.

Statistical analysis was done using a two-tailed Student *t* test; *p* < 0.05 was considered significant.The correlations between various categories were investigated.

Using the Pearson product-moment correlation coefficient, values less than 0.25 indicated a weak correlation, between 0.25 and 0.50 mild, between 0.50 and 0.75 moderate and greater than 0.75 good.

## Results

The points to be considered when assessing and analyzing the results in this study included the adequacy of the initial reduction, radiological union of the fracture and any loss of reduction or mal-union with any deformity, other possible complications such as loosening or osteolysis around the wires, myositis ossificans formation and the functional outcome of the injured elbow.

### Radiological results

**Fig. 5:**
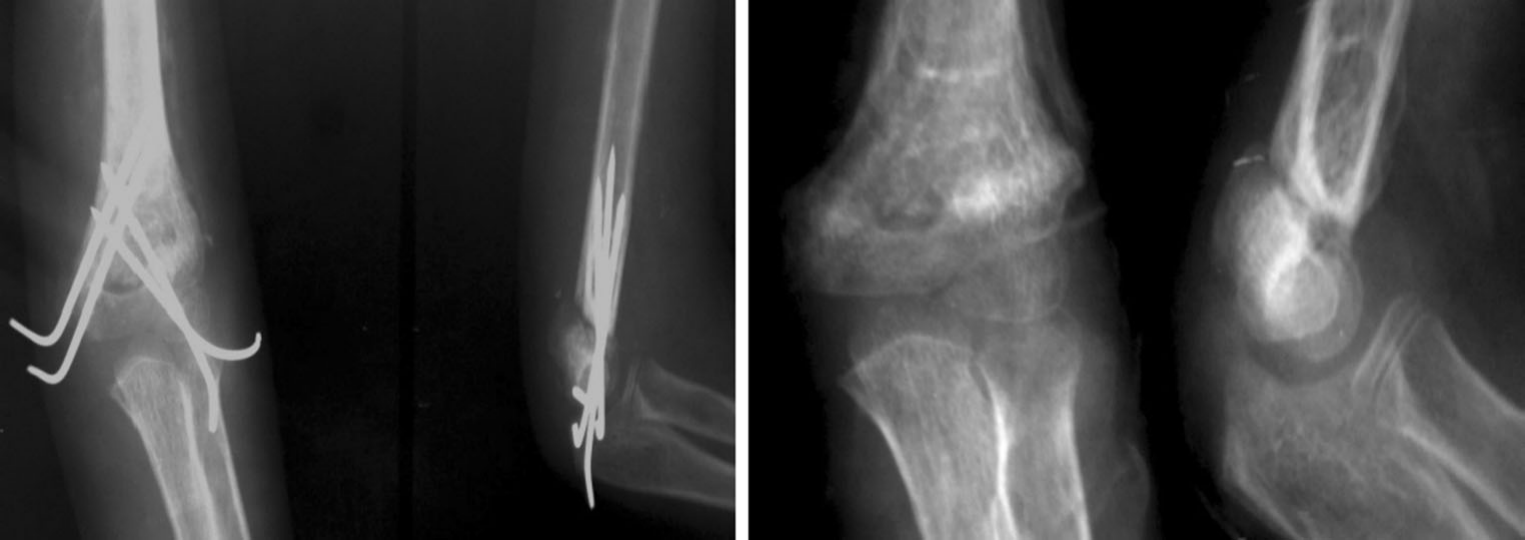
Fixation by four crossing wires and after complete union and removal of wires (patient 1)

**Fig. 6:**
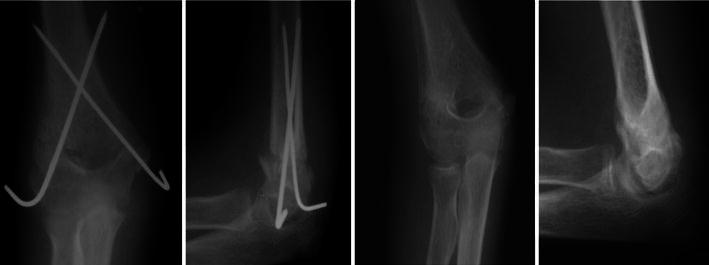
Fixation by two crossing wires and after complete union in excellent position and wire removal (patient 14)

**Fig. 7:**
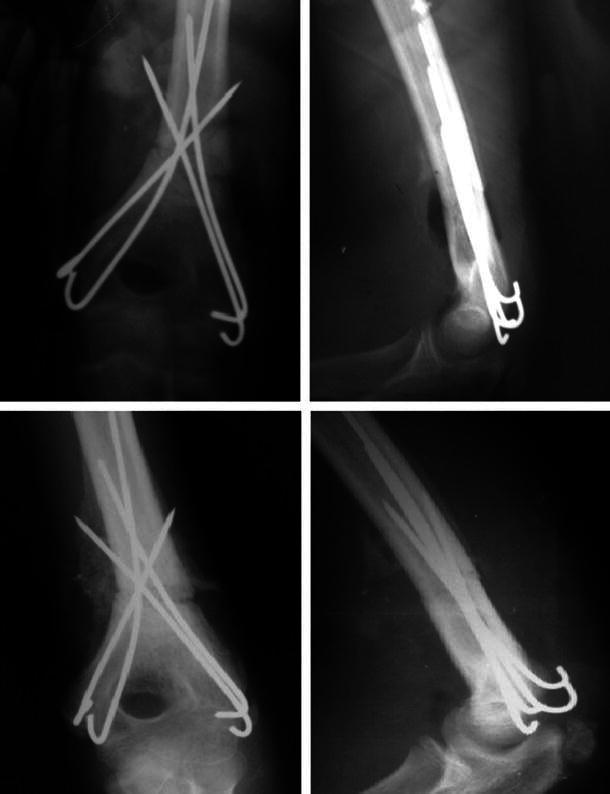
Fixation by four crossing wires immediately post-operatively and after union with visible callus (patient 8)

All fractures (100 % of patients) united (Figs. 5, 6, 7) in a mean duration of 7.2 weeks (range 5–10 weeks), as shown in Table 1. In one patient, the anterior humeral line, as a radiological sign of anatomical reduction, was not restored due to non-anatomical reduction in the lateral view X-ray (Fig. 5). In three patients, Baumann’s angle could not be measured radiologically in the antero-posterior view due to imperfect radiographic projections. Although in a number of patients (four) the anatomical radiographic parameters of the elbow (Baumann’s angle—anterior humeral line) were not measurable or not perfect, patients had no axial deviation or deformity.

**Table 1:** Characteristics of the presented patients

Cases	Age (years)	Type of fracture	Time needed for union (weeks)	Injury/surgery interval (days)	Surgery/full ROM interval (weeks)	Follow-up period (months)	Complications
1	10.00	Extension-type	10	27	Not fully restored	10	Re-fracture
2	6.00	Extension-type	5	16	10	17	Superficial pin track infection
3	9.20	Flexion-type	7	18	15	18	No complications
4	9.90	Extension-type	7	18	16	14	No complications
5	8.10	Extension-type	6	16	12	15	No complications
6	7.00	Extension-type	5	16	8	8	No complications
7	8.00	Extension-type	9	17	13	14	Superficial pin track infection
8	10.00	Lower 1/4 humerus	10	34	19	49	No complications
9	8.00	Extension-type	6	16	12	12	Superficial pin track infection
10	9.20	Extension-type	7	17	14	15	No complications
11	8.00	Extension-type	6	18	10	22	No complications
12	10.00	Extension-type	9	19	Not fully restored	17	Superficial pin track infection
13	9.00	Extension-type	6	17	14	13	Superficial pin track infection
14	11.00	Flexion-type	10	22	20	17	No complications
15	6.20	Extension-type	5	16	8	16	No complications
Mean ± SD	8.6 ± 1.3		7.2 ± 1.9	19 ± 5	13 ± 3.8	17 ± 9.4	

Variation in the union time could be due to different factors such as the site of fracture (supracondylar or lower humeral fracture), the degree of displacement and the need for much dissection, the presence of comminution, the different ages of the patients studied, and the time interval between fracture and ORIF. Statistical analysis of the results showed that the younger the patient (age) the faster the union (time needed for union), and the earlier the intervention (injury–surgery interval) the faster the union (time needed for union), as shown in Table 2.

**Table 2:** Correlation between the ages of the patients, the injury–surgery intervals and the time needed for union

Time needed for union	Pearson correlation	*P* value	Significance
Age	0.835	0.001	HS
Injury–surgery interval	0.749	0.001	HS

*HS* highly significant

The fracture type (extension or flexion), the direction of displacement (postero-lateral or postero-medial) and the number of wires (two or four) or the wires being hidden or protruding from the skin had no influence on the healing time.

Up to complete radiological union and removal of wires, there was no loss of reduction or secondary displacement, no mal-union, and no loosening or osteolysis around the wires. There was no myositis ossificans at the last follow-up (Table 1).

### Functional results

All patients and parents expressed appreciation and satisfaction with the outcome, especifically functional recovery, except for two patients. The time to regain the normal ROM ranged from 8 to 20 weeks with a mean of 13 weeks, as shown in Table 1.

**Fig. 8:**
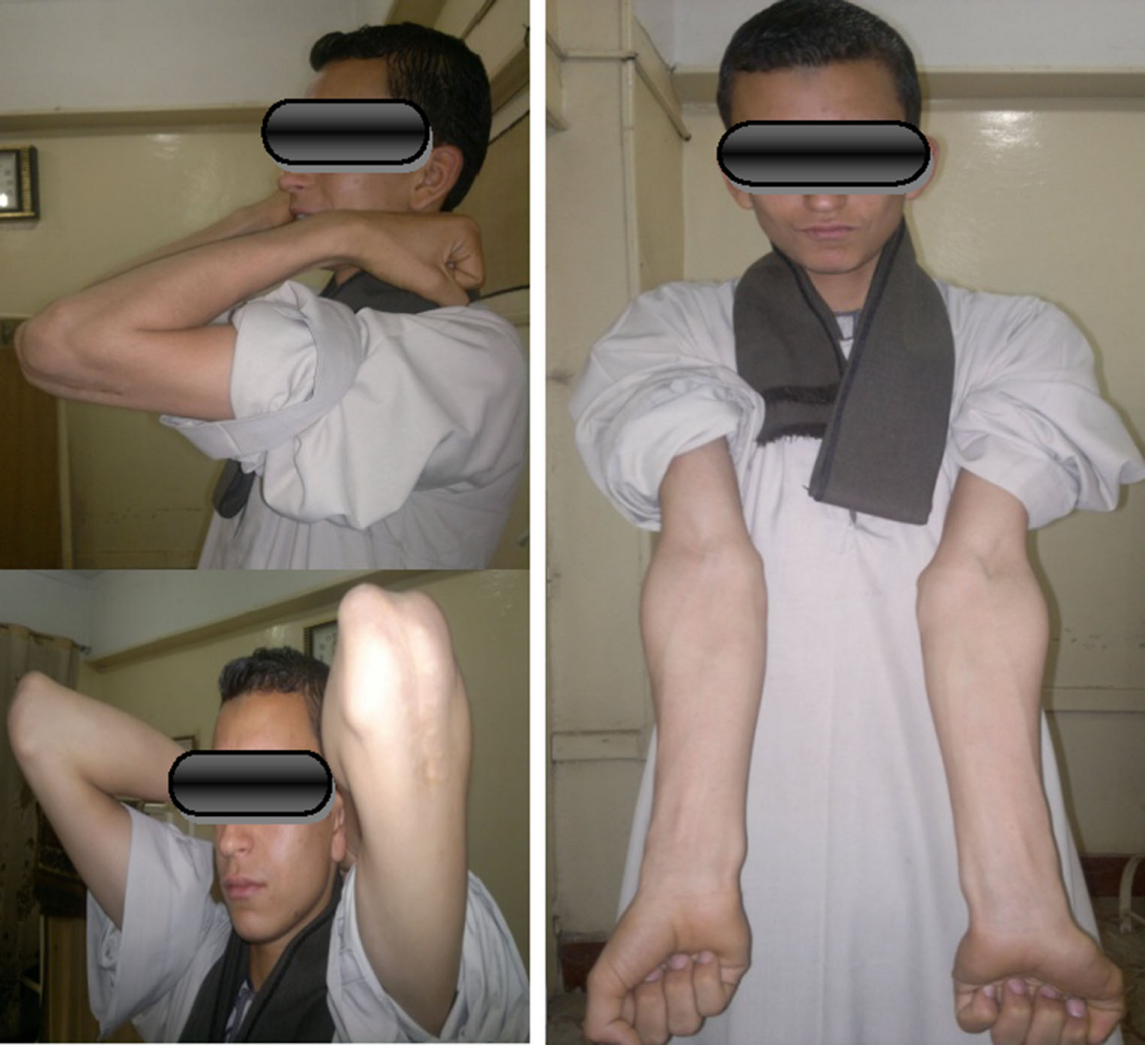
Full ROM of the elbow at the last follow-up 4 years post-operatively (patient 8)

**Fig. 9:**
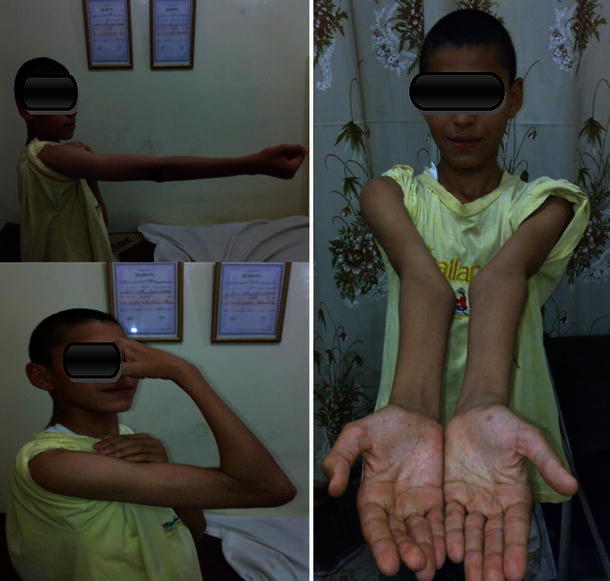
Nearly full ROM of the elbow at the last follow-up 1 year post-operatively (patient 14)

Excellent results were found in 13 patients (86.66 %) (Figs. 8, 9). Good results were found in two patients (13.33 %) at the last follow-up according to MEPI (Table 3). According to the Mark functional criteria, there was one patient with a fair result (6.66 %) (Table 4). The MEPI is much more forgiving than the criteria given by Mark et al., due to the difference in rating the amount of loss of motion in the elbow joint. This explains the difference in results using the two systems for evaluation.

**Table 3:** Results of the patients studied according to Mayo Elbow Performance Index (MEPI)

Result grade	Points	No. of cases		
Excellent	90–100	13		Mean MEPI 96.6
Good	75–89	2		–
		MEPI 86	MEPI 80	–
Fair	60–74	0		–
Poor	Below 60	0		–

**Table 4:** Results of the patients studied according to Mark criteria

Result grade	Loss of motion	Loss of carrying angle	Pain/neurovascular lesion	No. of cases
Excellent	None	None	None	13
Good	<20°	<10°	None	1
Fair	20–50°	10–20°	Minimal pain with excessive use No neurovascular lesion	1
Poor	>50°	>20°	Pain/neurovascular lesion	0

Five patients (33.33 %) developed superficial pin track infections that started 1 week after slab removal and at the beginning of active ROM; this was managed simply by pure alcohol and oral antibiotics, as shown in Table 1.

No patients had iatrogenic nerve injury or vascular insult. No patients developed deep wound infection or wire loosening or migration. No patients developed physeal arrest or deformity up to the last follow-up.

**Fig. 10:**
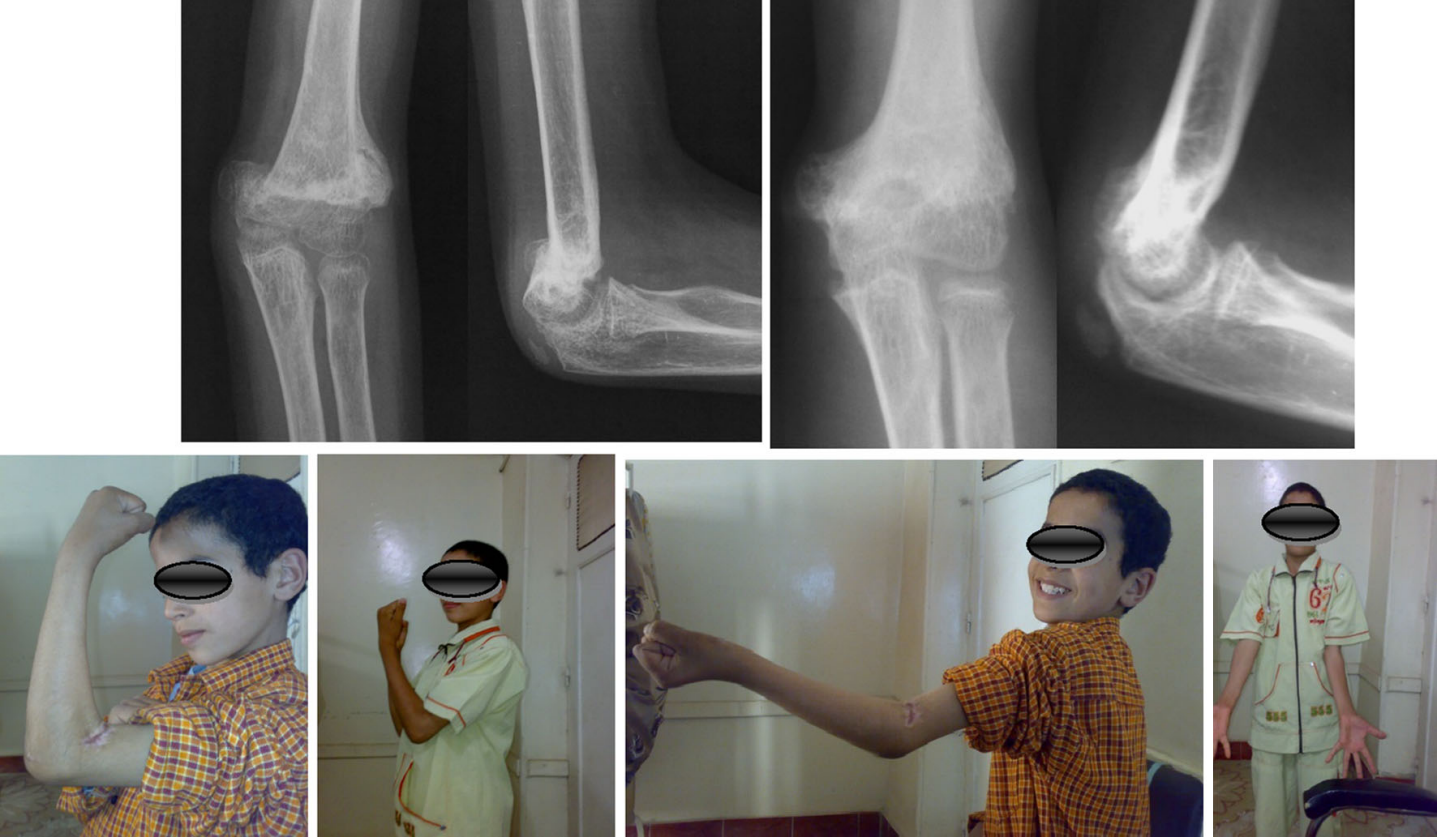
The patient with iatrogenic re-fracture after complete union and remodeling with loss of more than 30° of motion, denoting a fair result according to the criteria given by Mark et al. Full finger and wrist functions denoting spontaneous recovery of the previously affected radial nerve (patient 1)

Re-fracture occurred in one patient (6.66 %) with incomplete restoration of the ROM. In this patient, the time to union was about 10 weeks and after wire removal there was inadequate callus formation in the lateral view. The patient was again put in above elbow slab for another 2 weeks for protection. There was marked limitation of the ROM, mostly due to volar skin abrasions with contracted elbow flexors due to the prolonged immobilization period. The patient was referred for physiotherapy, and after 2 weeks presented with a swollen tender elbow. X-ray revealed re-fracture; he was put in a slab for 3 weeks, the slab was removed after the pain completely disappeared and X-ray showed dependable callus formation. He was sent back for gentle physiotherapy and followed up monthly until improvement. After 6 months, there was complete union and remodeling of the fracture to an accepted position denoted by the anterior humeral line cutting the capitulum as a radiological sign of good reduction with improved ROM (Fig. 10).

## Discussion

It is clear that the goal of treatment of any fracture is to obtain consolidation without complications.

According to the literature, children with neglected supracondylar humeral fractures are those who presented for treatment after 14 days of injury and had already started the biological process of healing with early callus formation.

There are various factors leading to delayed treatment following supracondylar humeral fractures in children. Inability to achieve a satisfactory closed reduction of the fracture due to continued swelling and/or skin problems is the main concern. The need for ORIF increases as the time to surgery increases. The rate of conversion to open reduction has been reported as ranging from less than 3 % to about 46 % [15].

Tiwari et al. [16] consider operative treatment the best option for such late-presenting fractures. In the past, open reduction led to concerns regarding elbow stiffness, myositis ossificans, unsightly scarring and iatrogenic neurovascular injury. However, several studies [17] have recently demonstrated a low rate of complications associated with open reduction. Some authors have demonstrated no correlation between stiffness and the type of surgical approach used, especially regarding the posterior approach [18].

All patients in this study presented after more than 2 weeks, with a mean duration between their presentation and the initial injury of 19 days (range 16–34 days).

A study by Lal and Bhan [19] included 20 children with delayed open reduction by means of a posterior approach for supracondylar humeral fractures. The delay time ranged from 11 to 17 days. In another study by Eren et al. [3] the average delay time was 6 days (range 2–19 days).

The average time for complete union in the current study was 7.2 weeks (range 5–10 weeks). In a study by Dehao et al. [20], all fractures healed within 6–8 weeks. The difference in healing time could be due to the fact that the mean age of the patients in the present study and the interval between injury and presentation for surgery were larger than in the study by Dehao et al. [20].

Nerve injuries associated with displaced supracondylar humeral fractures may be separated into those associated with the injury itself and those associated with treatment of the injury [21]. A literature review demonstrated iatrogenic nerve injury in 3.6 % of patients, with the ulnar nerve being involved in 81 % of these cases [22]. In the current study, and due to fact that the fixation was done after open reduction with exposure and identification of the ulnar nerve, there was no iatrogenic nerve injury and the two patients with radial and ulnar nerve involvement pre-operatively resolved spontaneously within 3 months post-operatively with no need for nerve conduction studies.

In this study, the time to regain the normal ROM ranged from 8 to 20 weeks with a mean duration of 13 weeks, with faster recovery in patients with less immobilization. In the study by Eren et al. [3], full functional recovery was achieved within 3 months in 29 patients (93.5 %), and there was no evidence of a correlation between duration of immobilization and delay in ROM recovery.

Regarding complications such as pin track infection, deep infection, compartment syndrome, mal-union and deformities, the results of the present study are comparable with other studies by Lal and Bhan, Dehao et al., Eren et al. and Tiwari et al. [19, 20].

Another study by Jason et al. [12] preserved triceps integrity and function using an extensor mechanism-on approach for fixation of distal humeral fractures. They documented that there is limited literature regarding elbow motion, functional outcomes and objective strength assessment following the extensor mechanism-on approach; although the age group and mode of fixation were different from that in the current study, the results could be matched regarding the functional recovery of the elbow.

Jason et al. [12] documented that open treatment of distal humeral fractures with an extensor mechanism-on approach results in excellent healing, a mean elbow flexion–extension arc exceeding 100°, and maintenance of 90 % of elbow extension strength compared with that of the contralateral, normal elbow.

In comparison with the other studies, the current study is characterized by an older age group of patients, a longer time interval of neglected treatment and a wide variety of cases including flexion and extension types of supracondylar fractures and also lower humeral fractures.

In conclusion, patients with neglected supracondylar fractures of the humerus are those who presented for treatment after 14 days of injury and in such neglected cases—especially those with early appearance of callus—there is no place for trials of closed reduction and percutaneous pinning. Finally, we can conclude that triceps-sparing approach isan easy, simple and safe approach for exposure and internal fixationof neglected supracondylar and distal humeral fractures in childrenwith excellent functional outcome.
